# Using Approximate Bayesian Computation to infer sex ratios from acoustic data

**DOI:** 10.1371/journal.pone.0199428

**Published:** 2018-06-21

**Authors:** Lisa Lehnen, Wigbert Schorcht, Inken Karst, Martin Biedermann, Gerald Kerth, Sebastien J. Puechmaille

**Affiliations:** 1 University of Greifswald, Zoological Institute and Museum, Applied Zoology and Nature Conservation, Greifswald, Germany; 2 NACHTaktiv – Biologists for Bat research GbR, Erfurt, Germany; University of Western Ontario, CANADA

## Abstract

Population sex ratios are of high ecological relevance, but are challenging to determine in species lacking conspicuous external cues indicating their sex. Acoustic sexing is an option if vocalizations differ between sexes, but is precluded by overlapping distributions of the values of male and female vocalizations in many species. A method allowing the inference of sex ratios despite such an overlap will therefore greatly increase the information extractable from acoustic data. To meet this demand, we developed a novel approach using Approximate Bayesian Computation (ABC) to infer the sex ratio of populations from acoustic data. Additionally, parameters characterizing the male and female distribution of acoustic values (mean and standard deviation) are inferred. This information is then used to probabilistically assign a sex to a single acoustic signal. We furthermore develop a simpler means of sex ratio estimation based on the exclusion of calls from the overlap zone. Applying our methods to simulated data demonstrates that sex ratio and acoustic parameter characteristics of males and females are reliably inferred by the ABC approach. Applying both the ABC and the exclusion method to empirical datasets (echolocation calls recorded in colonies of lesser horseshoe bats, *Rhinolophus hipposideros*) provides similar sex ratios as molecular sexing. Our methods aim to facilitate evidence-based conservation, and to benefit scientists investigating ecological or conservation questions related to sex- or group specific behaviour across a wide range of organisms emitting acoustic signals. The developed methodology is non-invasive, low-cost and time-efficient, thus allowing the study of many sites and individuals. We provide an R-script for the easy application of the method and discuss potential future extensions and fields of applications. The script can be easily adapted to account for numerous biological systems by adjusting the type and number of groups to be distinguished (e.g. age, social rank, cryptic species) and the acoustic parameters investigated.

## Introduction

The proportion of males (POM) in animal populations is of great interest to ecologists and conservationists, because sex ratios influence mating systems and effective population size, the latter being crucial for the maintenance of genetic diversity [1]. In species whose sex cannot be easily identified visually because they are elusive or nocturnal, or lack visible secondary sex dimorphism, other means of determining the POM are required. Molecular sexing can offer an alternative [2,3], but is usually time and cost-intensive, and often invasive due to the challenges arising from non-invasively collected samples, e.g. the need to process replicates of each sample and the associated increase in costs [3,4].

In species with vocal sexual dimorphism, these issues can be circumvented by acoustic sexing. In the last decades, passive acoustic monitoring has become a popular tool for collecting information about biodiversity [5], population densities [6,7], animal movement and behaviour [8], and how these factors are impacted by anthropogenic activities [9]. Both in terrestrial and marine environments, acoustic data have gained in importance [9,10] across taxonomic groups from insects [11], amphibians [12], reptiles [13], fish [14,15], and birds [16] to mammals [17–22]. Alongside species identity [21,23,24] acoustic data can encode information about sex [25–28], individual identity [25,29], body size, age, and social group [28,30–32] or geographic origin [33–35]. Sex identification based on calls, for example, has successfully been applied to 25 of 69 investigated bird species lacking external sex dimorphism [36]. Acoustic sexing was however less reliable or even impossible in many of the studied species due to varying degrees of overlap in acoustic characteristics of males and females. Such overlap, which is commonly encountered across taxonomic groups [36,37], strongly limits the scope of the application of acoustic data. Therefore, novel approaches are required that allow acoustic sexing and inferences of sex ratios despite such an overlap.

To investigate the possibility to infer sex ratios and to provide sexing methods from acoustic data, we use *Rhinolophus hipposideros* (*Rhip*) as a biological model showing acoustic sexual dimorphism with overlap between sexes [28]. This bat species is of high conservation concern: it is classified as near threatened in the European Union Red List and listed in the annex II and IV of the EU Habitats and Species Directive. Hence population monitoring is legally required. In *Rhip*, the reproductive output of colonies is usually estimated by dividing the number of juveniles by the number of adult females. The latter however cannot be easily determined from visual counts of adults in the colony, because most bat species, including *Rhip*, show no visible secondary sex dimorphism. As in many mammalian species, sex identification in bats mostly relies on catching individuals and inspecting their sexual organs. For regular monitoring, this approach is unsuitable due to the large time effort required and handling stress for the animals, especially for vulnerable or threatened species. Current estimates of reproductive output therefore usually assume all adults present in the colony to be females. Males have however been reported to be present in *Rhip* maternity colonies in the past [38–40]. Zarzoso-Lacoste & Jan *et al*. [3] further demonstrated that the POM in maternity colonies can be substantial and importantly, differ between colonies. Traditional estimates are therefore prone to underestimate the number of offspring per female to an unpredictable degree and therefore call for alternative methods to be developed and tested.

The high calling rates of echolocating bats make them ideal for acoustic monitoring (e.g. [23,41]) and for developing methods of acoustic sexing. However, sexing based on simple call parameters such as the call frequency has not yet been possible, because these acoustic parameters overlap between sexes [28,42,43]. Hence, an approach that would allow a quick and simple determination of sex ratios in free-ranging populations despite such an overlap would greatly benefit monitoring programs and ecological studies. While different mixture model approaches exist to address this situation (cf. section “Screening for and testing available approaches”), we did not encounter an approach allowing reliable inference of the POM for overlapping ranges of acoustic parameters. As a consequence, we use Approximate Bayesian Computation (ABC) to infer sex ratios from passively recorded calls in a species with vocal sex dimorphism, where acoustic value ranges of males and females overlap. The developed novel approach moreover allows the investigation of spatial or temporal changes in sex composition, and the assignment of sex to calls. We additionally develop a simpler approach that only considers calls outside the overlap zone. The performance of all methods is tested with simulated data. We additionally validate the reliability of acoustic sex ratio determination with empirical data (recorded echolocation calls) from four molecularly sexed *Rhip* colonies.

All methods are implemented in the provided R-scripts (S1 and S2 Files). Users do not need to have programming experience if their study system meets the assumptions of the currently implemented approach, because the adjusted priors are the only input required. More experienced users can change additional settings to relax various assumptions and extend the approach to other study systems. Consequently, our approach has a wide range of applications in ecology and conservation biology.

## Materials and methods

### Screening for and testing available approaches

We assessed the list of R-based cluster analyses and finite mixture modelling approaches available on CRAN (https://cran.r-project.org/web/views/Cluster.html) for their suitability concerning our study question. The maximum-likelihood (ML) based package mclust [44] was considered a promising candidate and its performance was tested (see S3 File for details) with the simulated data described in the section “Simulated data set”. mclust performed relatively poorly in determining the POM, especially for low sample sizes, i.e. 100 or 200 calls (S1 Fig). The mclust package was therefore judged unsuitable for reliable estimation of the POM from acoustic data in *Rhip* colonies, where datasets of fewer than 200 calls are not uncommon. Other ML approaches were not tested as they did not meet the requirements of ratio inference from univariate, normally distributed data.

A major advantage of Bayesian approaches compared to ML approaches is the specification of priors [45] by the user, which permits the algorithm to draw on existing information, hence potentially greatly increasing performance. In our study system, the value ranges of male and female mean peak frequencies are known, providing information that can be used as priors to improve performance in the estimation of POM. Existing Bayesian clustering approaches however do not allow the specification of uniform, or flat, priors where all possible values within the range are equally likely *a priori*. To overcome this issue, we developed an ABC approach that incorporates both the required feature of ratio inference from univariate, normally distributed data and the flexibility to define uniform priors.

### Inferring sex ratios with ABC

The approach we developed is based on an ABC framework inferring the most likely parameters given some observed distribution by comparing summary statistics of the observed data to summary statistics of simulated datasets. In our case, the main parameter investigated was the POM in a given dataset (set of echolocation call recordings from many individuals). The characteristics of the simulated datasets are described in the section “Simulated data set” below. To generalize our approach and to allow for some uncertainty in prior knowledge in acoustic parameters for both sexes, we included three additional parameters: the mean peak frequency of each sex separately (two parameters) and the standard deviation (sd) of peak frequency of each sex. The latter was assumed to be equal for both sexes, and hence was represented by a single parameter, an assumption that can be relaxed. Based on our own observations (I.Karst, W. Schorcht, M. Biedermann) and published data on our focal species [28,46–48], the following parameter priors (with uniform distribution) were used: POM: 0–1, mean peak frequency of males: 104–107 (kHz), mean peak frequency of females: 108–111 (kHz), sd of peak frequency: 0.8–1.2 kHz. We used two additional constraints stating that the mean peak frequency of males was at least 2.5 kHz, but not more than 5 kHz lower than the mean peak frequency of females [47]. The comparisons between observed and simulated datasets used the following summary statistics a) median of peak frequencies, b) mean of peak frequencies, c) standard deviation of peak frequencies (all irrespective of sex), d) Kolmogorov-Smirnov distance between the observed and simulated distributions of peak frequencies.

We used the adaptive population Monte-Carlo ABC algorithm developed by Lenormand *et al*. [49]. This algorithm was preferred over others as it was designed to minimise the number of models necessary to reach a given quality of the posterior distribution and was the only one providing reasonably good estimates when sex ratios were severely imbalanced (data not shown). The ABC algorithm was implemented via the EasyABC package [50] in R version 3.4.2 [51]. Unless otherwise stated, the input arguments used for the algorithm were 1000 for the initial number of simulations (*nb_simul*), 0.4 for the proportion α of best-fit simulations to update the tolerance level at each step (*alpha*), and 0.01 as the stopping criterion (*p_acc_min*). The values of alpha and *p_acc_min* can influence the quality of the posterior approximation, and the present values were chosen as they performed best in simulations exploring all 42 different combinations of values between both parameters tested (alpha: 0.4, 0.5, 0.6, 0.7, 0.8, 0.9; *p_acc_min*: 0.001, 0.005, 0.01, 0.02, 0.03, 0.04, 0.05; see S2 Fig). The 95% highest density intervals (HDI) of the estimates obtained via the ABC approach were computed using the ‘hdi’ function from the ‘HDInterval’ package [52].

### Simulated data set

Simulations used in the testing phase of the ABC algorithm and to perform the ABC analyses were implemented via a custom R script (S1 and S2 Files). For a given total sample size (here, number of calls) and POM, we calculated the number of data points (calls) from females and males, respectively, and simulated normally distributed peak frequency data for each sex separately. We assumed the ratio of male to female calls to be equal to the ratio between the number of males and the number of females. The samples sizes used were 100, 500, 1000, 2500, 5000, and 10000; the POM ranged from 0 to 1 in incremental steps of 0.05, with 100 replicates per parameter combination. The mean peak frequency was either set by the user to create test datasets or was automatically chosen by the ABC algorithm. For the test datasets, the following parameter values were used: male mean peak frequency = 106 kHz, female mean peak frequency = 109 kHz, and sd of peak frequency = 1 for each sex. When running the ABC algorithm, priors were used as defined in the above section. The normal distribution was used as it fitted well to the distribution of peak frequencies observed in *Rhip* [47].

### Inferring sex ratios by excluding the overlap zone

**Fig 1:**
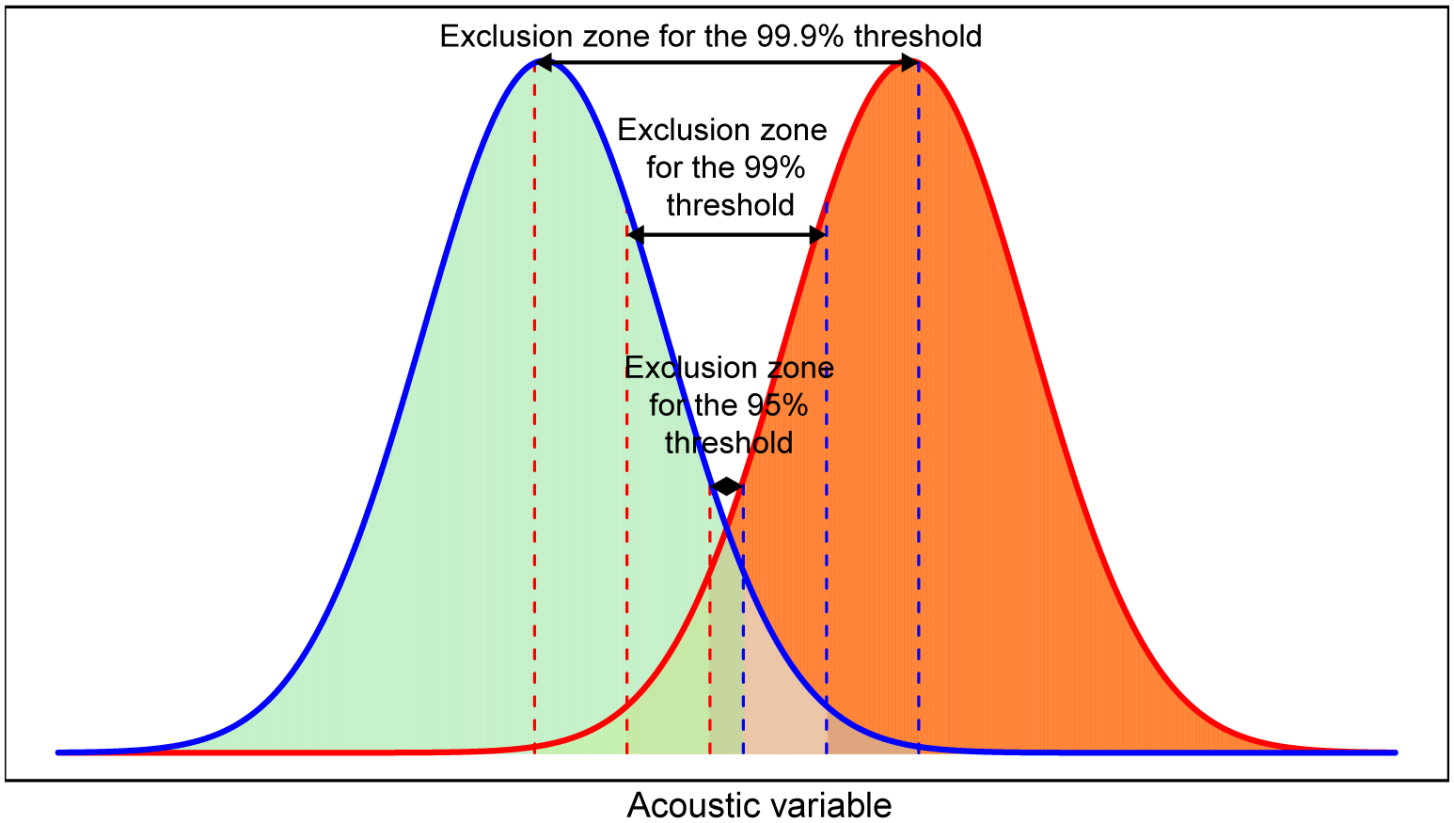
Illustration of the exclusion method. The x-axis represents the acoustic variable of interest (e.g. peak frequency) with different, but overlapping, distributions in males (blue) and females (red); both represented as a probability density function. Red/blue dashed lines depict the investigated lower/upper boundaries for the exclusion zone, with different stringency thresholds used to define the overlap zone.

Mirroring approaches from species without overlapping acoustic value ranges, we developed a second method determining sex ratios that solely considered calls that can be classified with high confidence as male or female. The POM was then simply the proportion of male calls in the dataset. Calls falling within the overlap range were therefore excluded in this method (exclusion method). To investigate trade-offs between confidence levels and reductions in sample size, we tested the performance of this method using different thresholds for the exclusion zone. For simplicity, we always considered males to call lower than females, so that the exclusion zone encompassed the right tail of the distribution of male calls and the left tail of the distribution of female calls. In the most stringent case (widest exclusion zone), the lower boundary of the exclusion zone was set to the frequency that was lower than the frequency of 99.9% of all female calls, and the higher boundary to the frequency that was higher than the frequency of 99.9% of male calls (Fig 1). The less stringent approaches used thresholds of 99 and 95%.

This exclusion method used normally distributed data with the mean of the acoustic parameter for each sex and the sd given by the ABC estimation. It can also be applied without using the ABC method but then the value of these parameters must be provided by the user based on prior knowledge of the system. We therefore examined how the accuracy of the assumed parameter values affected the estimated POM. For this purpose, we explored the effect of assuming mean peak frequency values that were up to 3 kHz higher or lower than the true (simulated) value. For simplicity, we did not investigate errors in the assumed sd of peak frequencies and the difference in mean peak frequency between sexes, hence our calculations are most likely underestimating the true error. The 95, 99 and 99.9% thresholds were calculated as explained above based on the user provided values of mean peak frequencies of males and females. Calculations were carried out for male proportions between 0 and 1 in incremental steps of 0.05.

### Methods’ performance

The performance of the different methods in estimating the POM was evaluated by computing the root-mean-squared error (RMSE):

$$RMSE\;=\;\sqrt{\sum_{k=1}^{R}{\left(p_{i}-P\right)}^{2}/R}$$

where *p*_*i*_ is the estimate of the simulated (≙true) proportion of males *P* for the *i*th data set (*i* = 1, 2, …, R).

### Two-step approach for small data sets

As the precision of estimates obtained with the ABC method diminished with decreasing sample size (see Results), precise sex ratio estimation might be precluded for small datasets (i.e. low numbers of calls). This applies to studies that are interested in temporal or spatial changes in the POM, e.g. over a month or between different habitat patches, thus requiring estimates on a daily basis or for restricted spatial areas. Using simulated data, we explored the use of a two-step ABC procedure whereby we first estimated the acoustic parameters for each sex (mean and sd) across the whole dataset (e.g. for a whole month). A second step used the 95% highest posterior density interval of these estimates as new priors to run the ABC procedure for a subset of these data (e.g. each day separately). This two-step approach assumes that the sex-specific mean of acoustic parameters does not change in time/space within the dataset considered, i.e. the mean peak frequencies and sd do not change for example from day to day (time) or between two foraging patches (space). We investigated the performance of the two-step procedure using simulated datasets mimicking a POM increase over time in steps of 0.05 from 0 to 1 (≙21 steps, each representing a certain time period). The number of calls per period was kept constant. Simulations were carried out for 25, 50 and 100 calls per period so that the total number of calls per dataset was 525, 1050 and 2100, respectively. A total of 100 simulated datasets was used per parameter combination.

### Inferring the sex per recording

The ABC framework does not provide information about the sex of each data point (here, call). Nevertheless, the information on acoustic parameters and the POM it provides can be subsequently used to probabilistically assign a sex to each data point. To do so, we calculated the likelihood ratio of a call being from a male versus a female as the conditional probability of a call being from a male given its frequency divided by the conditional probability of a call being from a female given its frequency. Following Bayes’ theorem, the conditional probability of observing a male given the peak frequency can be written as:

P(Male|Freq)=P(Freq|Male)xP(Male)/P(Freq)

The conditional probability of observing a female given the peak frequency can be written as:

P(Female|Freq)=P(Freq|Female)xP(Female)/P(Freq)

The likelihood ratio (LR) can thus be reduced to:

LR=P(Freq|Male)xP(Male)/P(Freq|Female)xP(Female)

Calls with a likelihood ratio >1 or <1 were considered as being from males or females, respectively.

Using simulated datasets, we investigated the proportion of calls assigned to the correct sex using this probabilistic approach. Simulated datasets and simulation parameters were as detailed in ‘Simulated data set’ and the POM ranged from 0.1 to 0.9 in steps of 0.1. To investigate the performance of the approach with varying levels of overlap between male and female call distributions, differences of 1, 2, 3, 4, and 5 kHz between male and female mean peak frequencies were considered. Sample sizes of 25, 50, 100, 500 and 1000 calls were investigated with 1000 replicates per parameter combination (225,000 simulated datasets).

### Empirical dataset

The empirical data set consisted of *Rhip* echolocation calls recorded in four maternity colonies in Thuringia, Central Germany, in the summers of 2015 and 2016 (see S1 and S2 Tables for details). Accessing the roosts was approved by local nature conservation authorities (permit Jena AV09_AGO7_17). One automatic recording device (Anabat SD2 bat detector, Titley Scientific) was positioned inside each of the four studied roosts and directed towards the entrance (ca. 3 m away) to record bats entering between 1:00 am and 6:00 a.m. using a zero-crossing division ratio of eight. We used the Anabat CFCread program (Titley Scientific) to split the continuous data into recordings, i.e. sequences of calls considered to be emitted by one animal passing the detector (for details see S4 File: What is a recording in our study?). Recordings were then filtered via the software AnaLook version 4.2.n to include only *Rhip* calls (S5 File). Subsequently, calls with an average frequency (Fmean) below 100 kHz were removed as they were outside the value range of the call parameter for adult *Rhip* and to exclude social calls, which have a lower frequency [53]. Fmean and sd were calculated for each recording. The mean of Fmean calculated over all calls for each recording was considered representative of the acoustic parameter (i.e. peak frequency) of the emitter.

### Molecular sexing

Bat faeces were non-invasively collected in the studied colonies during the summers of 2015 and 2016 (see Table 1). Newspapers were spread on the ground beneath the roosting sites and faeces were collected approximately 10–20 days later (Table 1). Upon collection, faeces were stored in plastic boxes (1 per colony) pre-filled to 1/3 with absorbing silica-gel beads to prevent DNA degradation until analysis [54,55]. Animals were not touched during the sampling, and accessing the roosts was approved by local nature conservation authorities (permit Jena AV09_AGO7_17). DNA extraction and amplification, as well as multilocus genotyping were carried out as described by Zarzoso-Lacoste & Jan *et al*. [3], but employing centrifugation instead of a vacuum pump during DNA extraction (see S6 File for a detailed protocol). We also used the same bioinformatics pipeline, with slight modifications: In the current study, all relative fluorescence unit (RFU) peaks corresponding to sex specific alleles were visually re-inspected and validated by plotting the RFUs for the appropriate marker with the aid of the R-package Fragman [56]. Furthermore, we applied a different rule for the peak of the Y-linked allele to be accepted: it was counted only if the corresponding peak was present in all three replicates and higher than the peak of the X-linked allele in at least one replicate. An exception was made in the rare case where a multilocus genotype (MLG) was found to be identical to another MLG in all loci but the sex specific one. In that case, the Y-linked allele was also counted if it was missing in one replicate and if the corresponding peak was not higher than the peak of the X-linked allele. In general, only MLGs with missing data at no more than two loci were kept, provided that the peaks at the sex specific locus were unambiguous.

**Table 1:** Proportion of males and peak frequencies of the studied colonies. The proportion of males was estimated with the genetic (Gen.), acoustic ABC (ABC), and acoustic 99.9% exclusion methods. The 95% highest density interval (HDI) is presented for parameters estimated via the ABC approach. Peak frequencies were estimated with the acoustic ABC approach.

	Samples			Proportion of males			ABC estimate of the mean peak frequency in kHz	
ID	a)	b)	c)	Gen.	ABC, mean (HDI)	99.9	Male, mean (HDI)	Female, mean (HDI)
Thu22	144	138	78	0.22	0.21 (0.19–0.23)	0.19	105.9 (105.72–106.08)	109.1 (109.02–109.15)
Thu26	168	153	83	0.63	0.63 (0.57–0.69)	0.67	105.9 (105.78–106.09)	108.6 (108.39–108.86)
Thu47	40	38	19	0.32	0.37 (0.34–0.39)	0.34	105.5 (105.33–105.57)	108.8 (108.70–108.88)
Thu35	40	39	23	0.26	0.37 (0.32–0.41)	0.38	105.0 (104.73–105.27)	108.3 (108.14–108.56)

a) number of genetic samples.

b) number of samples that provided multilocus genotypes (MLGs) of sufficient quality (see Materials and methods for details).

c) number of unique MLGs.

## Results

### Estimation of acoustic parameters and sex ratios with ABC

**Fig 2:**
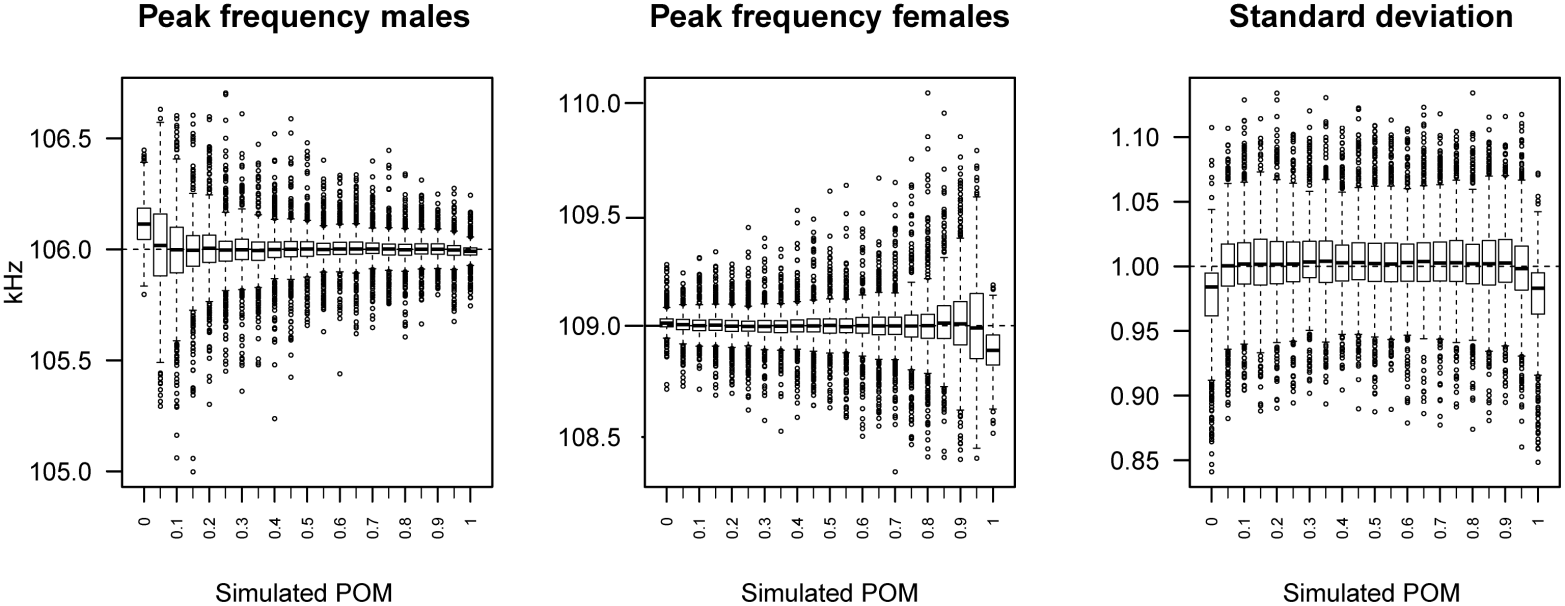
Mean peak frequencies of males and females and within-sex standard deviation of peak frequency. Same sd for both sexes, estimated via ABC across simulations (i.e. including all tested sample sizes: 100, 500, 1000, 2500, 5000, 10000; with 100 simulated data sets for each of the 126 combinations of a given proportion of males and sample size).

**Fig 3:**
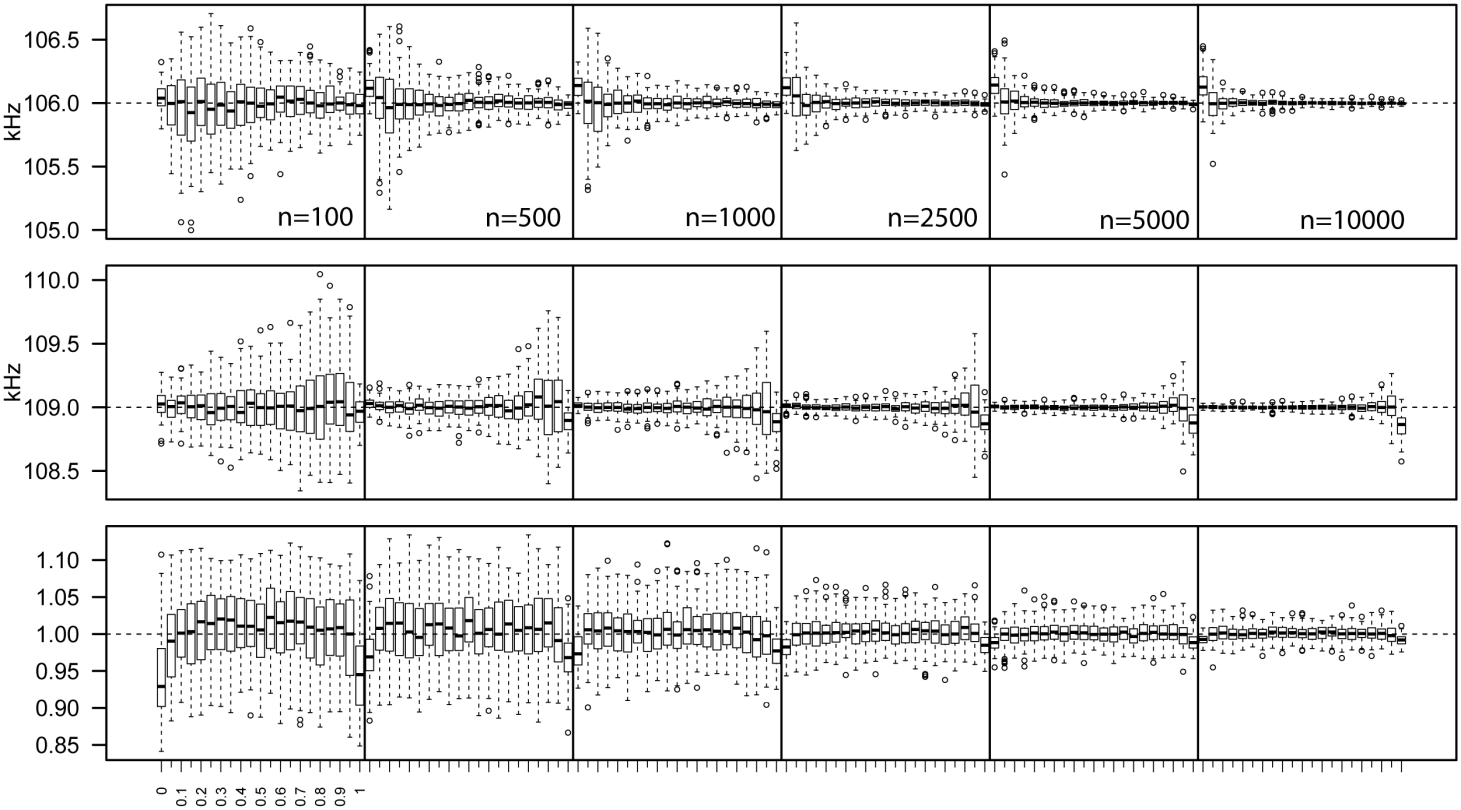
Mean peak frequency of males (top) and females (middle) and the within-sex (equal for males and females) standard deviation of calls (bottom). Estimated via ABC for the same datasets as in Fig 2, but plotted separately for the six different sample sizes (represented by blocks).

The estimated male and female mean peak frequencies matched the simulated values. Inaccurate estimates were obtained however if the proportion of the focus sex was zero (Figs 2 and 3). Both precision and accuracy increased with sample size (Fig 3). The estimated sd of call frequencies was slightly underestimated when only one sex was present (Figs 2 and 3). Generally, there was a slight upward bias in the estimated sd that disappeared with increasing sample size (Fig 3).

**Fig 4:**
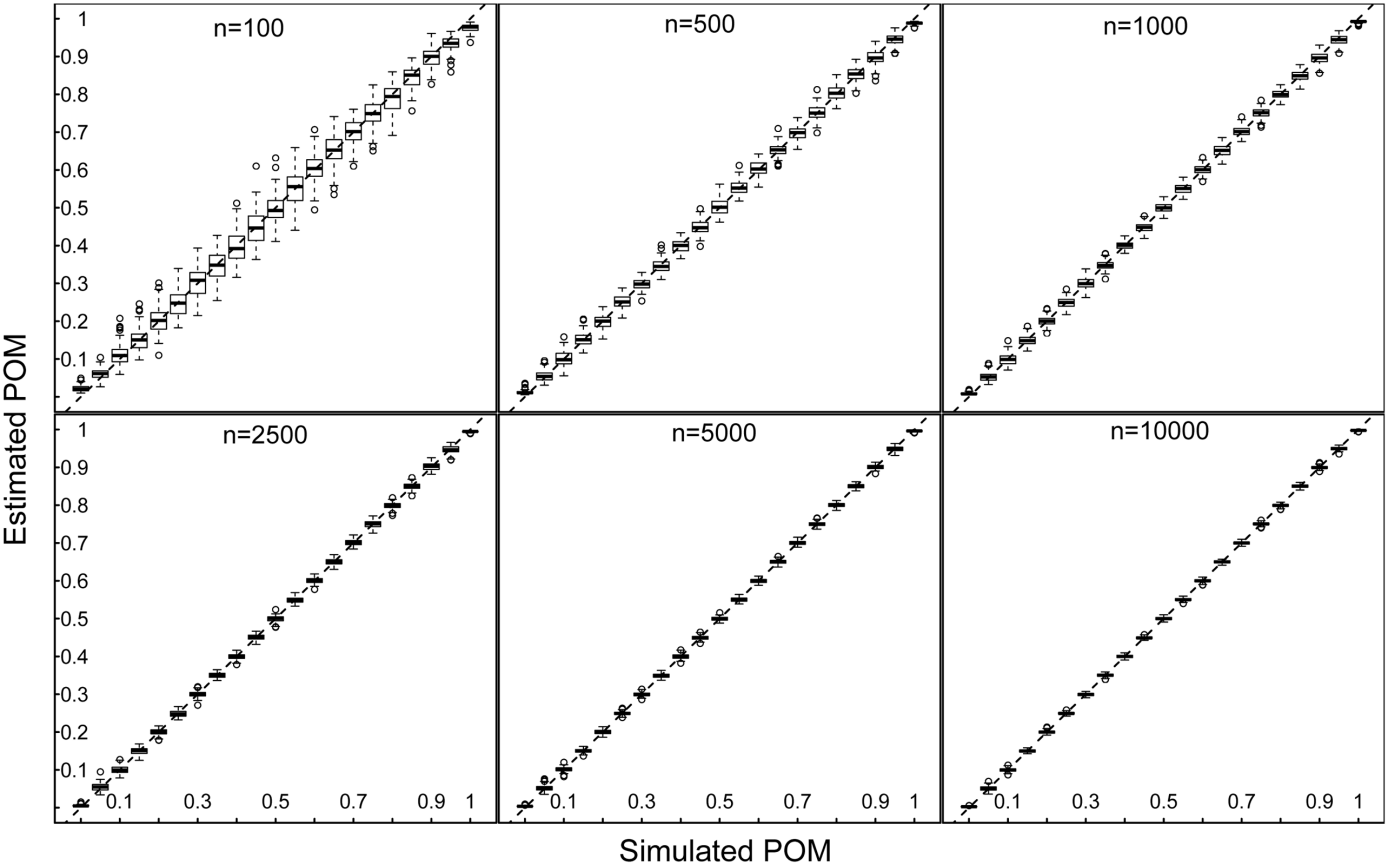
Proportion of males estimated via the ABC method versus the simulated (true) value. For 100, 500, 1000, 2500, 5000, and 10000 calls (same datasets as Figs 2 and 3). The dashed line depicts a perfect match between simulated and estimated values.

**Fig 5:**
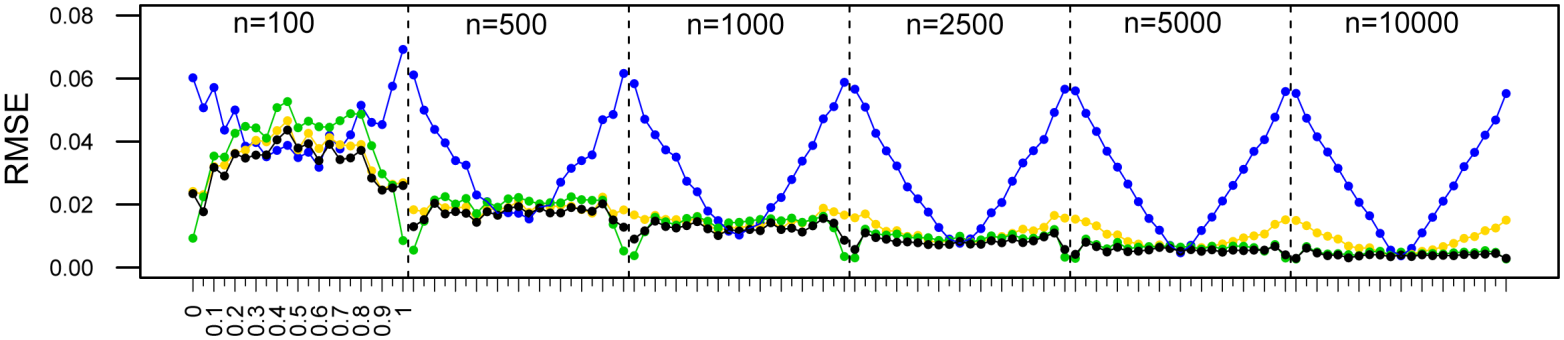
Root mean square error (RMSE) for different sex ratios estimated via the exclusion method using thresholds of 95% (blue), 99% (yellow), and 99.9% (green), and via the ABC approach (black). Blocks correspond to different sample sizes as indicated. The X-axis represents the simulated proportion of males (from 0 to 1 in steps of 0.05, same datasets as Figs 2–4).

POM values estimated via ABC showed a high degree of concordance with the real values in the simulated data set for all sample sizes (Fig 4). Both precision and accuracy increased with sample size (Figs 4 and 5). The RMSE did not exceed 0.05 for the lowest sample size (100 calls), and dropped to less than 0.02 when increasing the number of calls to 500 (Fig 5). Further increases in the number of calls resulted in even lower RMSEs for all sample sizes tested, but beyond 5000 calls the improvement became marginal (Fig 5).

For the four sampled *Rhip* colonies (empirical datasets), mean peak frequencies were inferred to be between 105.0 and 105.9 for males, and between 108.3 and 109.1 for females (Table 1). The POM estimates ranged between 0.21 and 0.63 depending on the colony, differing from the genetically determined one by 0 to 0.11.

### Two-step ABC approach

**Fig 6:**
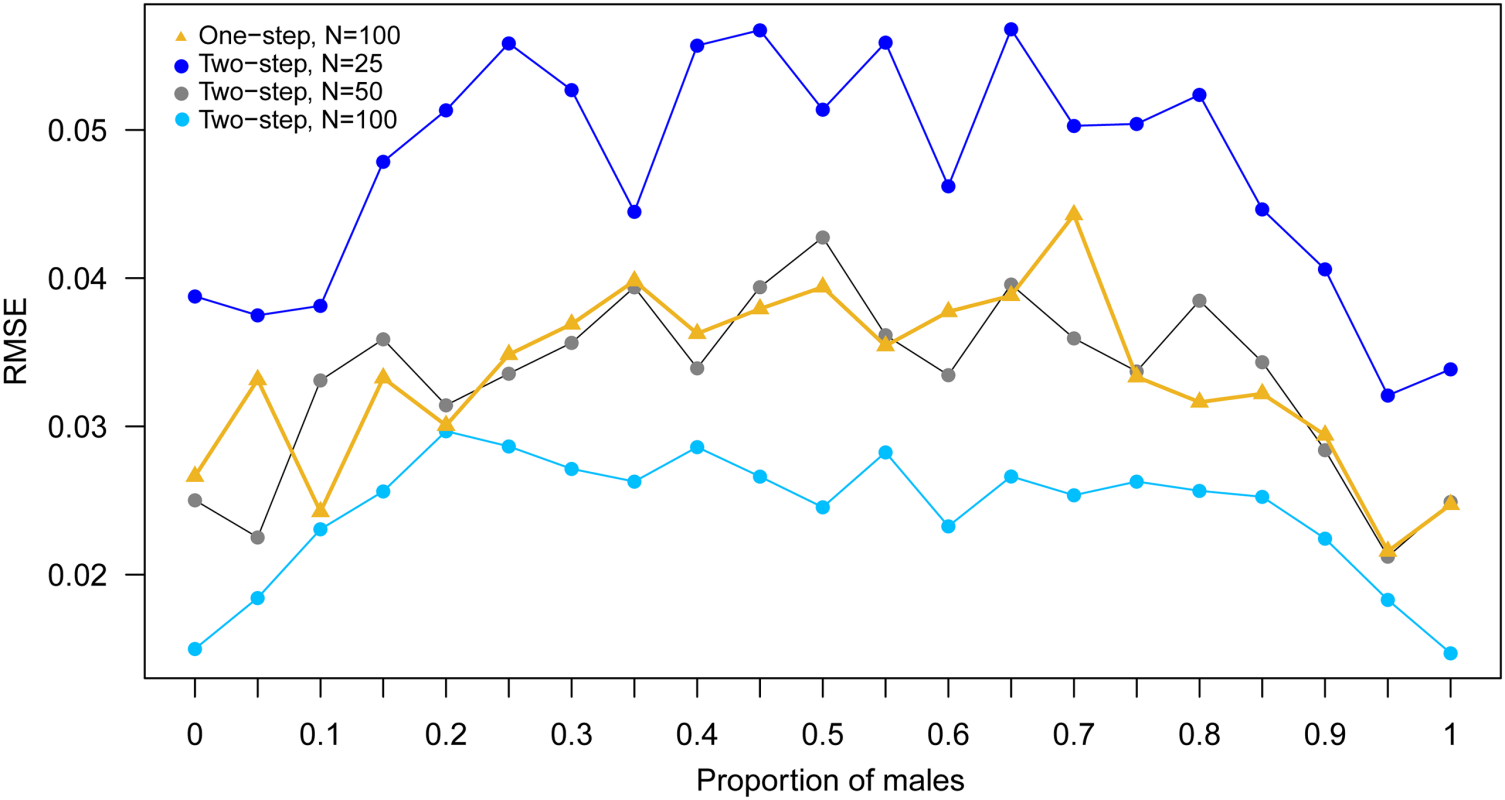
Comparison of RMSEs obtained with the one-step versus the two-step ABC method.

Applying a two-step ABC procedure improved the performance of POM estimates compared to an approach where acoustic parameters (male and female peak frequencies and sd) were estimated separately for each subset (Fig 6). RMSEs below 0.04 were obtained for datasets of 50 calls, which matched the performance of the one-step approach but with a sample size reduced by half (Fig 6).

**Fig 7:**
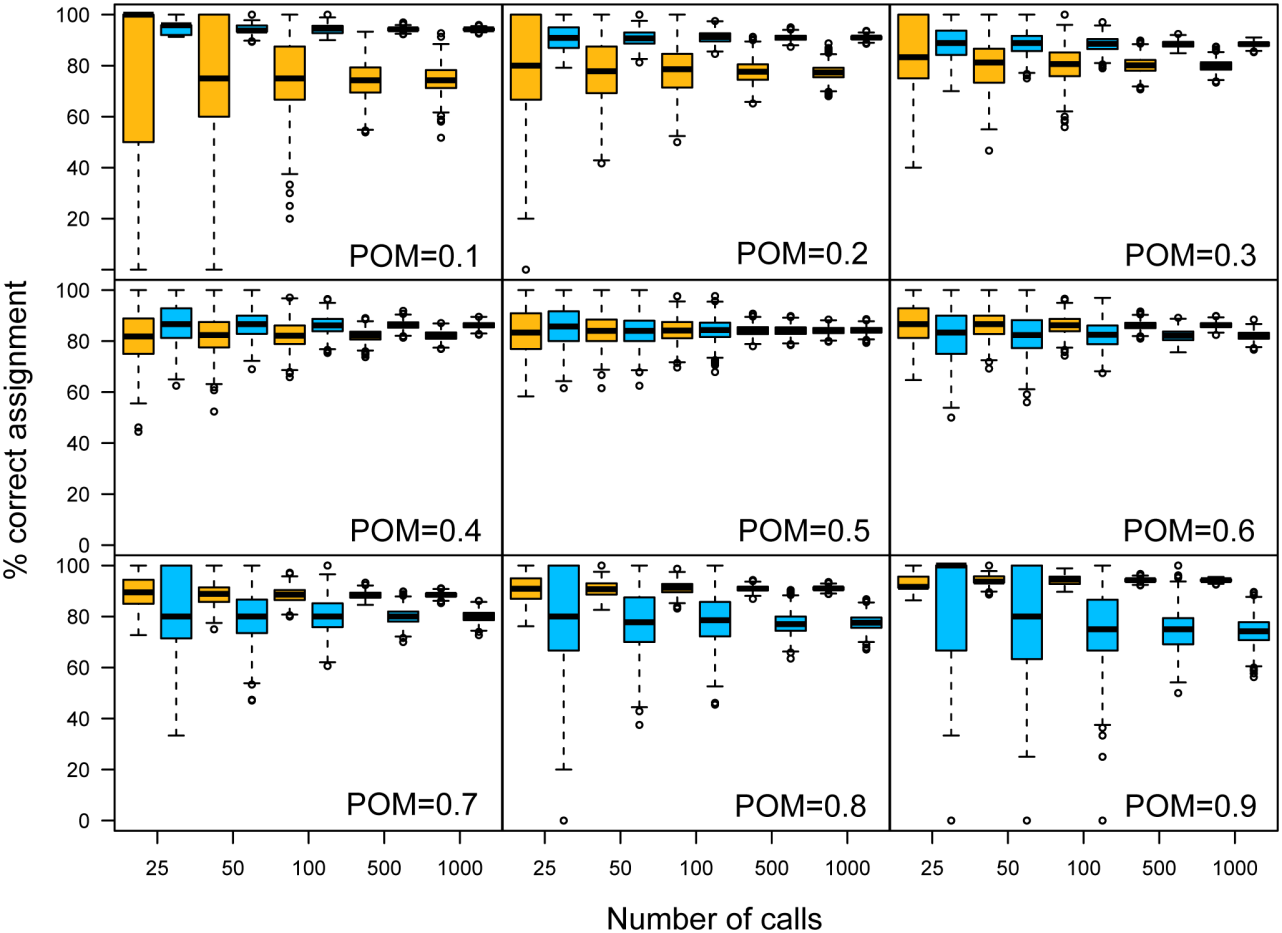
Percentage of calls correctly identified as male (yellow) or female (blue). In this example, simulated mean peak frequencies of both sexes differed by 2 kHz and the number of calls per dataset ranged from N = 25 to N = 1000. Values are presented for 1000 simulated datasets per considered proportion of males (0.1 to 0.9 in steps of 0.1) and number of calls.

**Fig 8:**
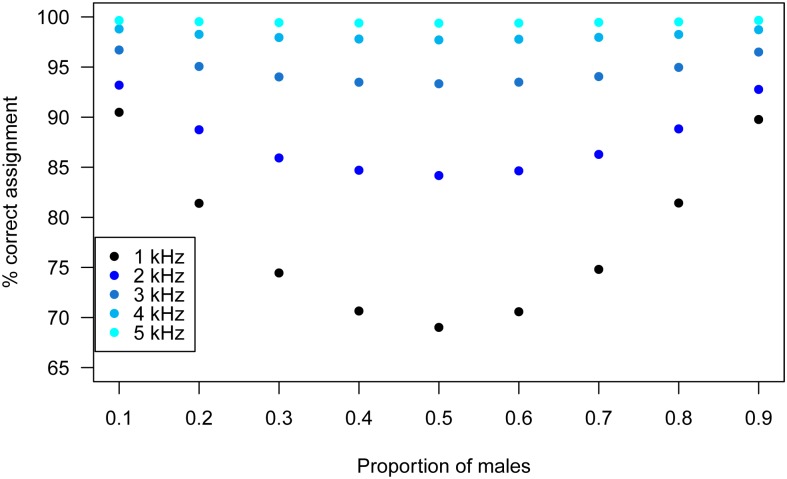
Influence of the proportion of males on the global percentage of correct sex assignment of calls when male and female mean peak frequencies differ by 1–5 kHz. Values for different sample sizes (cf. Fig 7) and sexes are combined.

The proportion of calls assigned to the correct sex using the probabilistic approach was influenced by the difference in mean peak frequency between sexes, the number of calls as well as the POM (Table 2; Fig 7). For minor overlaps between sexes (i.e. 4–5 kHz difference in mean), the method performed extremely well (> 98% calls correctly assigned; Table 2). When the overlap increased, the method still performed relatively well, with e.g. 78% calls correctly assigned when the overlap zone comprises 95% of calls in the dataset (1 kHz difference, N = 1000 calls; Table 2). The variance across datasets, but not the mean correct assignment, decreased with increasing number of calls. The POM affected the results in two ways; first, the more common sex had a larger proportion of correct assignment (Fig 7); second, the overall proportion of correct assignments was higher for more unbalanced sex ratios (Fig 8).

**Table 2:** Identification of the caller’s sex from simulated datasets. The difference between male and female simulated mean peak frequencies (Δ) was between 1 and 5 kHz. The number of calls per dataset ranged from N = 25 to N = 1000. Values presented correspond to the percentage (mean and sd) of individuals assigned to the correct sex out of 9,000 simulations (1000 simulations per considered proportion of males going from 0.1 to 0.9 in steps of 0.1). The percentage of calls within the overlap zone between sexes is also presented as a measure of overlap (mean and sd).

	Mean (sd) % correctly assigned					Mean (sd) % overlapping				
Δ	N = 25	N = 50	N = 100	N = 500	N = 1000	N = 25	N = 50	N = 100	N = 500	N = 1000
5	99.5 (1.4)	99.5 (1.0)	99.5 (0.3)	99.5 (0.3)	99.5 (0.2)	0.2 (1.2)	0.3 (1.3)	0.5 (1.5)	1.7 (2.1)	2.6 (2.6)
4	98.1 (2.7)	98.2 (1.9)	98.1 (0.7)	98.1 (0.7)	98.1 (0.6)	1.7 (4.6)	2.7 (4.7)	4.4 (5.2)	11.0 (7.1)	15.0 (7.9)
3	94.5 (4.6)	94.6 (3.4)	94.7 (2.5)	94.6 (1.6)	94.6 (1.4)	9.3 (11.7)	13.8 (11.8)	20.0 (12.5)	36.8 (13.2)	45.0 (12.8)
2	87.7 (7.0)	87.7 (5.4)	87.7 (4.5)	87.7 (3.5)	87.7 (3.4)	30.4 (19.9)	40.5 (18.6)	51.0 (17.2)	71.7 (12.3)	78.7 (10.1)
1	78.1 (10.2)	78.1 (8.9)	78.1 (8.3)	78.1 (7.8)	78.1 (7.7)	61.0 (22.2)	72.7 (17.0)	81.2 (12.8)	93.1 (5.4)	95.6 (3.6)

### Estimation of sex ratios via the exclusion method

**Fig 9:**
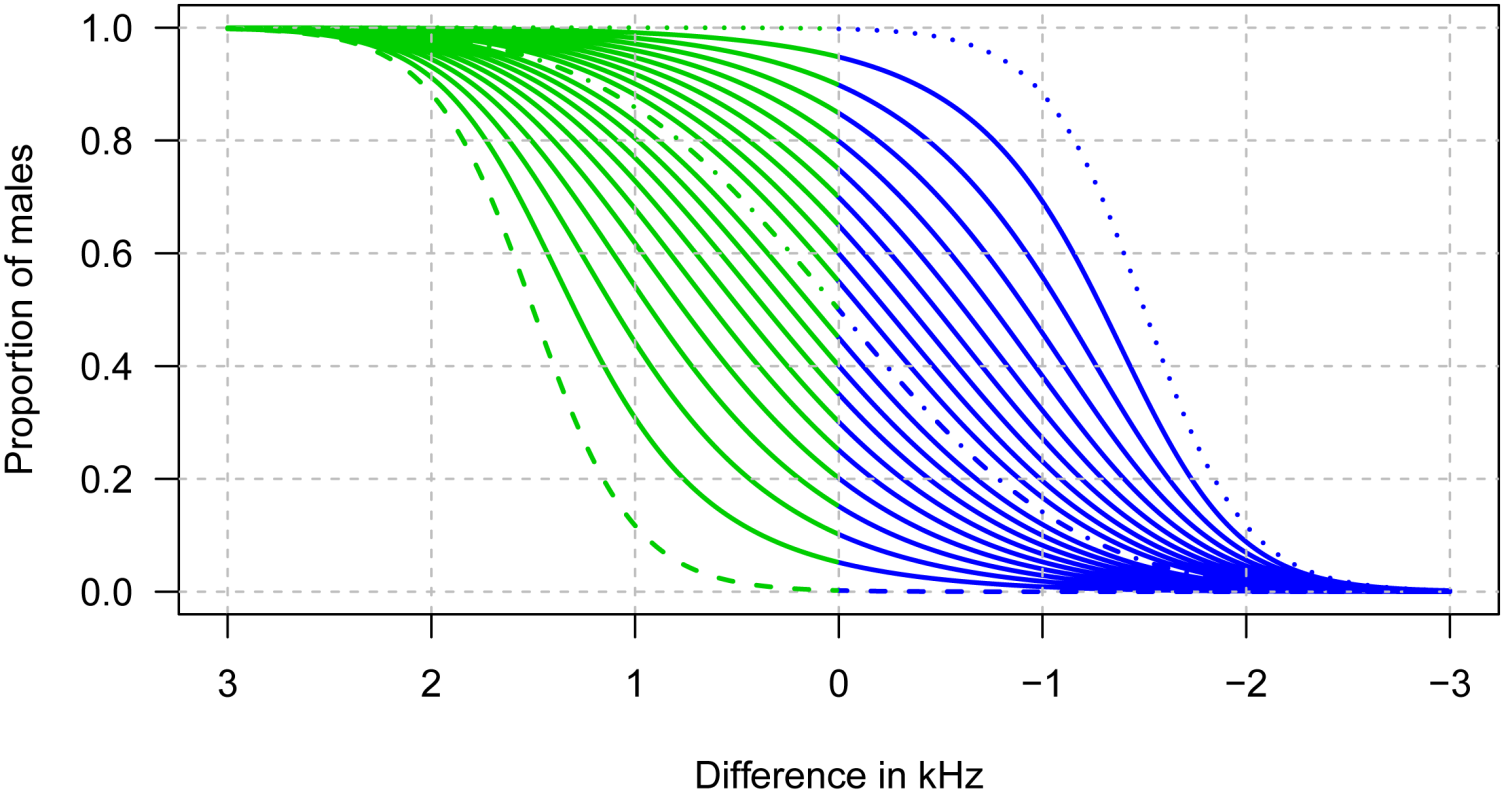
Impact of the accuracy of the assumed mean peak frequencies on the performance of the exclusion method (99.9% threshold). The x-axis shows the difference between the assumed and true mean peak frequency of male calls. Green lines represent an overestimation of the proportion of males, blue lines an underestimation. The dashed, broken, and dotted lines depict sex ratios estimated for different assumed male mean peak frequencies when the simulated POM is 0, 0.5, or 1, respectively.

Sex ratios estimated via the exclusion method varied in their performance in relation to the thresholds considered for the overlap zone. The 99% and particularly 95% approach performed as well as the ABC method for rather balanced sex ratios only, but resulted in higher RMSEs for unbalanced ones (Fig 5). A threshold of 99.9% yielded similar RMSEs as the ABC approach (Fig 5). However, the exclusion method was highly sensitive to the accuracy of the prior information, i.e. assumed mean peak frequencies of males and females (Fig 9). The sensitivity was dependant on the difference between the true and assumed mean peak frequency, the sign of this difference and the true POM.

The POM estimated via the 99.9% exclusion method in the four sampled *Rhip* colonies was between 0.19 and 0.67 depending on the colony. These estimates differed from the corresponding ABC results by 0.01 to 0.04, and from the genetically determined sex ratios by 0.02 to 0.12 (Table 1).

## Discussion

We have developed and tested two methods to infer the sex ratio of groups of vocalizing animals where acoustic parameters of the signals overlap between males and females. The newly developed Bayesian (ABC) approach infers male and female mean peak frequencies and the according sd as well as the proportion of males (POM). These inferences can then be used to separately analyse subsets of the data that correspond e.g. to certain time periods of interest and/or to probabilistically assign a sex to single data points (e.g. calls). Finally, the inferred acoustic parameters can be used to define the exclusion zone for a second, simpler approach based on the exclusion of calls from the overlap zone.

### Acoustic parameter estimation via ABC

The estimate of the mean call frequency for a specific sex becomes more precise and accurate with higher sample sizes and/or a higher proportion of that sex (Figs 2 and 3). However, in the absence of males, male mean peak frequency values are systematically overestimated, because calls from the left tail of the female distribution are treated as male calls. Vice versa, female mean peak frequencies are systematically underestimated in the absence of females (Fig 2). Consequently, call parameter estimates should be interpreted cautiously when inferred sex ratios are very imbalanced (i.e. when the estimated proportion of one of the sexes is less than 0.05), especially for low sample sizes (i.e. less than 500 calls).

Our method of estimating call parameters has broad applications beyond the determination of sex ratio. It could for example benefit studies interested in ecogeographic variation in call parameters, which has been observed in various taxa [35,47,57,58]. Our method favours the quick, relatively cheap, and non-invasive collection of a large amount of data, thus enabling researchers to compare acoustic traits across many sites. Our method could also be used to study other biological groups beyond sexes. For example, in some species, acoustic features differ based on social rank (e.g. [30,59]), age, or body size (e.g. [26,28,31,32,60]). Cryptic species could also be investigated if they differ (at least on average) for some acoustic parameter, which is often the case [61–67]. Our ABC approach can be used to determine the values which are characteristic for vocalizations of subgroups and to estimate their ratio in abundance. Even though the method has been developed for obtaining ratios for two groups, it can be extended to any number suitable for the study question.

### Sex ratio estimation

We confirm the robustness of the ABC and to some extent the exclusion method for sex ratio estimation with simulated data sets of varying sample size. Obtaining larger numbers of calls clearly improves precision and accuracy, but even for as few as 100 calls, errors are minor for the Bayesian approach (Fig 5). For our simulated datasets, the 99.9% exclusion method is slightly less accurate than the Bayesian approach for small sample sizes, but performs better compared to the 95% and 99% exclusion approaches (Fig 5). The exclusion method is very straightforward, providing an advantage for practitioners (easily applicable and very quick). However, the mean peak frequencies of males and females (and their sd) used in this approach must be known with very high accuracy to obtain reliable POMs (Fig 9). If this is not the case, we strongly recommend using the ABC method instead.

We additionally tested the ABC and exclusion methods with empirical data. For three of the four *Rhip* colonies, the POM determined from recorded calls is nearly equal to the ratio obtained via molecular sexing (Thu22, Thu26, Thu47; Table 1). These results provide clear evidence that the reliable estimation of the POM from acoustic recordings demonstrated via simulation also applies to empirical datasets. Our method thus can be used to obtain sex ratios and hence, the number of females, providing more reliable information to calculate the number of offspring per female in *Rhip* colonies from passive acoustic monitoring data. An overestimation of the POM via ABC compared to molecular sexing was observed in the remaining colony (Thu35; Table 1). Theoretically, the molecular sexing approach could suffer from a bias introduced by different sampling probabilities of males and females, e.g. because they prefer different hanging sites or due to differences in roost fidelity [3]. Due to the very good match between molecular and acoustic sexing for the other three colonies we however rather suspect an underrepresentation of some individuals in the recorded calls. The roost of this colony has two entrances, but only one was acoustically monitored. Hence, if more females than males preferred the unmonitored entrance over the one where recordings were made, their calls might be missing or less frequent in the data set, resulting in an overestimation of males. Therefore, we strongly recommend considering sex-specific or individual behavioural differences in the study design of future applications. Importantly, our method assumes a similar acoustic detection probability for individuals belonging to different groups of interest. If for example males call louder or more often than females, they will be overrepresented in the dataset and their proportion will be overestimated. If such differences in detection probability are well quantified for the study species/context, they can be corrected for when simulating datasets within the ABC method. For our target species, the calls are used for orientation and hence likely to be emitted equally often and at similar intensity by individuals of both sexes. Nevertheless, for a successful application of our method to other contexts or species, similarities in signal detection probability should be considered and discussed.

### Two-step sex ratio determination for data subsets

We have developed and tested a method to track changes in the POM via a two-step ABC approach. With this method, sex ratios can be estimated quite reliably even for relatively low numbers of acoustic signals, e.g. short time periods such as days, or small spatial scales such as single foraging/commuting sites. This allows the detection of temporal or spatial patterns in the POM, which could provide interesting insights into the flexibility of social organisation and behavioural differences of males and females in habitat use [68]. The method could be used to compare sex ratios obtained for groups of migrating animals passing a recorder station at different time periods (temporal) or to compare data simultaneously collected from different stations (spatial), for example. Migratory birds are a particularly promising target for such an approach, as acoustic signals even of flying flocks can be used for sex determination in certain species [69]. Furthermore, existing acoustic data from monitoring programs (e.g. [70]) could be used to test for within-species (or between—species, in the case of cryptic species) temporal/spatial segregation or habitat preferences. Similarly to the estimation of acoustic parameters, this temporal/spatial resolution method can also be applied to biological groups other than sex. To ensure accurate estimates, the choice of the temporal/spatial scale will depend on (1) the number of calls that can be obtained and, (2) the confidence in the dataset to meet the assumptions of the method (i.e. the mean of acoustic parameters for each group remaining constant through time/space).

### Inferring the sex per recording

We have additionally established a method to probabilistically assign a sex to single acoustic signals based on the acoustic parameters and the POM inferred from the ABC approach. While not error-free at the individual level, our method provides a good strategy for detecting overall patterns of sex-specific behaviour whose temporal/spatial resolution exceeds by far even that of the two-step Bayesian method. As the sex assignment of a single acoustic signal is based on the overall probability of signals being emitted by a male or female within the studied group, which in turn depends on the sex ratio, the two-step ABC method should be used prior to assigning a sex to single signals to confirm temporal/spatial homogeneity in sex composition, or to identify appropriate time periods/spatial scales over which the POM is stable in the population.

### Technical considerations for future applications

We have developed a method to infer the relative ratio in abundance of two (or potentially more) groups of animals with partially overlapping value ranges of acoustic parameters. The choice of parameters and distributions used in our investigation was driven by the biological model used to empirically test the method. Our work therefore only covers a small range of parameter combinations compared to what can be encountered in empirical datasets across taxa. For example, normally distributed data are ubiquitous in biology and we expect many datasets to conform to this distribution. Nevertheless, the use of other distributions is very straightforward within the proposed framework which can therefore accommodate datasets with a wide spectrum of distributions. The distributions can be any probability distribution with a known mathematical function (e.g. normal) but also any empirical distribution obtained by fitting an empirical dataset, offering within a single tool greater flexibility than with conventional maximum likelihood or conventional Bayesian models. The range of estimated parameters used in the ABC approach can be customised depending on the dataset. In our case, we used the same sd for male and female peak frequencies but these can be decoupled if needed. Similarly, we selected four summary statistics to compare the simulated and the observed dataset in the ABC procedure but different statistics could be used. We chose the mean, median and sd of peak frequencies (irrespective of sex) and the Kolmogorov-Smirnov statistic between the observed and simulated dataset as these are summarising well the variations observed in different distributions. However, the summary statistics can be easily and quickly customised to improve performance if needed.

When developing the algorithm, Lenormand *et al*. [49] found that smaller *alpha* and *p_acc_min* improved the quality of the posterior approximation but also increased the number of runs and hence time for completing the analysis. The authors recommended to use *alpha* = 0.5 and *p_acc_min* between 0.01 and 0.05 depending on the desired level of convergence. Our simulations show a limited influence of the values chosen for those parameters, probably because of the simplicity of our datasets and quick convergence. Nevertheless, it remains important to test the influence of these parameters and select those providing the most accurate estimates based on simulated datasets mimicking the empirical datasets of interest. More generally, we recommend that prior to studying sex ratio differences in empirical datasets, simulations mimicking those empirical datasets should be carried out to investigate the reliability and limitations of the method.

Based on previous investigations of lesser horseshoe bats returning to their roost from foraging trips (data not shown), we chose the spatial arrangement of the recording device and the temporal resolution of the recordings in a manner that limited the probability of two or more individuals being recorded simultaneously. While the simultaneous recording of multiple individuals cannot be ruled out, the good agreement between the ABC estimates of POM and the independent non-invasive genetic estimates suggests that those occurrences are not problematic in our empirical dataset. This suggests that when two animals are unlikely to be recorded simultaneously, one could simply use the mean peak frequency per recording (as done in our study). An alternative would be to filter out those recordings or eventually, one could use the peak frequency of each call instead of the mean peak frequency per recording. We did not investigate this strategy in our empirical dataset because the prevalence of recordings with high sd (suggesting simultaneous recording of more than one individual) was low (data not shown). However, this issue should be investigated in other organisms/situations where such recordings might be common, and especially when the acoustic parameters of calls of the emitter are altered in the presence of other individuals [71,72].

Bats were recorded when entering the roost. Exiting bats were not recorded to avoid multiple recordings per individual introduced by individuals going in and out or circling around the entrance. Small entrances maximise the probability that individuals approach the recording device from similar angles and at similar speed, leading to comparable Doppler shift on all recordings. Setting up the recording device in a more open environment (e.g. foraging grounds or commuting routes) might result in individuals approaching from different angles and recordings being obtained from animals either approaching or going away from the microphone, possibly leading to more ambiguous datasets. Although not tested here, choosing a microphone with high directionality might limit the problem though at the cost of less recordings being collected. Other non-mutually exclusive strategies might be to use the minimum value of the peak frequency per recording (instead of the mean), which is likely to represent the call emitted when the individual is going away from the microphone. Datasets collected in different situations might benefit from different data pre-processing steps prior to sex ratio estimation and we encourage users to explore different options (cf. also the above paragraph). Filtering should also be performed when different types of acoustic signals (e.g. echolocation versus social calls) are present in a dataset. Furthermore, in its current form, the developed ABC approach is based on the assumption that the detection probability is equal for both groups whose ratio shall be inferred. This deserves special attention because noncompliance with this assumption could lead to biases.

## Conclusions

The methods developed here will allow scientists and applied conservationists to investigate ecological questions dealing with the specific behaviour of groups of individuals in greater detail, provided that acoustic di-/polymorphism exists between the focal groups. These can comprise individuals that differ in an externally cryptic trait of interest, e.g. sex, species affiliation, age cohort, size or weight class, or social rank. We present a toolbox that combines the inference of acoustic parameters and sex ratio via ABC with downstream applications. The latter can be used to detect changes in group composition over time/space, or to assign single acoustic signals to one of the groups. To allow a broad field of application, we describe the conditions that must be fulfilled in order to apply our approach to other study systems and provide suggestions on how to overcome some challenges that may arise. The approach is based on acoustic data, which in many species can be acquired more easily than close-up morphological or genetic data, thus providing a non-invasive, time-efficient, and relatively low-cost approach to explore ecological traits that differ between groups of animals.

## Supporting information

S1 Fig RMSEs for different sex ratios estimated via mclust. Black line: RMSE with the number of groups determined by the model. Red dotted line: RMSE with the number of groups set to two by the user. Sample size is from n = 100 to 500 (in steps of 100), as indicated above the graph. Blue circles: RMSE obtained with the ABC approach.
(TIF)

S2 Fig RMSEs of estimates obtained for different parameter combinations of the ABC algorithm. RMSEs of estimates obtained with a sample size of 5000 calls for different parameter combinations of the ABC algorithm and different sex ratio; a) POM = 0, b) POM = 0.25, c) POM = 0.5, d) POM = 0.75 and e) POM = 1. The values of *p_acc_min* and *alpha* used are presented above and below the graphs respectively. Consistently low root mean square error (RMSE) was obtained for *nb_simul* = 1000, *alpha* = 0.4, *p_acc_min* = 0.01. Black: ABC, blue: 95% exclusion method, yellow: 99% exclusion method, green: 99.9% exclusion method.
(TIF)

S1 Table Sampling period and location of the four *Rhip* colonies investigated. (PDF)

S2 Table Empirical acoustic data. Fmean: call frequency (out of Analook). Colo: colony name. Rec.No: Recording number.
(CSV)

S1 File R-script for ABC analysis with simulated data. (R)

S2 File R-script for ABC analysis with empirical data. (R)

S3 File Testing the performance of the mclust package for estimating the proportion of males. (PDF)

S4 File What is a recording in our study? (PDF)

S5 File Filter used for Analook processing. abf file for import into Analook.
(ABF)

S6 File Modified protocol for extraction of DNA from bat faeces with the Macherey Nagel NucleoSpin^®^ Plant II extraction kit. (PDF)

## References

[1] Wedekind C. Managing population sex ratios in conservation practice: How and why? Topics in Conservation Biology. Rijeka, Croatia: InTech; 2012 pp. 81–96. http://www.intechopen.com/books/topics-in-conservation-biology/managing-population-sex-ratio-why-and-how

[2] Morinha F, Cabral JA, Bastos E. Molecular sexing of birds: a comparative review of polymerase chain reaction (PCR)-based methods. Theriogenology. 2012;78: 703–714 22704393 10.1016/j.theriogenology.2012.04.015

[3] Zarzoso-Lacoste D, Jan P-L, Lehnen L, Girard T, Besnard A-L, Puechmaille SJ, et al Combining noninvasive genetics and a new mammalian sex-linked marker provides new tools to investigate population size, structure and individual behaviour: an application to bats. Mol Ecol Resour. 2018; 18: 217–228. doi: 10.1111/1755-0998.12727 29058809 10.1111/1755-0998.12727

[4] Boston ESM, Puechmaille SJ, Scott DD, Buckley DJ, Lundy MG, Montgomery WI, et al Empirical assessment of non-invasive population genetics in bats: comparison of DNA quality from faecal and tissue samples. Acta Chiropt. 2012;14: 45–52. doi: 10.3161/150811012X654259

[5] Harris SA, Shears NT, Radford CA. Ecoacoustic indices as proxies for biodiversity on temperate reefs. Methods Ecol Evol. 2016;7: 713–724. doi: 10.1111/2041-210X.12527

[6] Marques TA, Thomas L, Martin SW, Mellinger DK, Ward JA, Moretti DJ, et al Estimating animal population density using passive acoustics. Biol Rev Camb Philos Soc. 2013;88: 287–309. doi: 10.1111/brv.12001 23190144 10.1111/brv.12001 PMC3743169

[7] Kloepper LN, Linnenschmidt M, Blowers Z, Branstetter B, Ralston J, Simmons JA. Estimating colony sizes of emerging bats using acoustic recordings. R Soc Open Sci. 2016;3: 160022 doi: 10.1098/rsos.160022 27069667 10.1098/rsos.160022 PMC4821278

[8] Wrege PH, Rowland ED, Keen S, Shiu Y. Acoustic monitoring for conservation in tropical forests: examples from forest elephants. Methods Ecol Evol. 2017;8: 1292–1301. doi: 10.1111/2041-210X.12730

[9] Laiolo P. The emerging significance of bioacoustics in animal species conservation. Biol Conserv. 2010;143: 1635–1645. doi: 10.1016/j.biocon.2010.03.025

[10] Thomas JA, Moss CF, Vater M. Echolocation in bats and dolphins. Chicago: The University of Chicago Press; 2004.

[11] Chesmore ED, Ohya E. Automated identification of field-recorded songs of four British grasshoppers using bioacoustic signal recognition. Bull Entomol Res. 2004;94: 319–330. doi: 10.1079/BER2004306 15301697 10.1079/ber2004306

[12] Pellet J, Schmidt BR. Monitoring distributions using call surveys: estimating site occupancy, detection probabilities and inferring absence. Biol Conserv. 2005;123: 27–35. doi: 10.1016/j.biocon.2004.10.005

[13] Colafrancesco KC, Gridi-Papp M. Vocal sound production and acoustic communication in Amphibians and Reptiles In: Suthers RA, Fitch WT, Fay RR, Popper AN, editors. Vertebrate Sound Production and Acoustic Communication. Springer International Publishing; 2016 pp. 51–82.

[14] Gannon DP. Passive acoustic techniques in fisheries science: A review and prospectus. Trans Am Fish Soc. 2008;137: 638–656. doi: 10.1577/T04-142.1

[15] Ladich F. Sound communication in fishes. Wien: Springer-Verlag Wien; 2015.

[16] Wimmer J, Towsey M, Roe P, Williamson I. Sampling environmental acoustic recordings to determine bird species richness. Ecol Appl. 2013;23: 1419–1428. doi: 10.1890/12-2088.1 24147413 10.1890/12-2088.1

[17] Brigham RM, Kalko EKV, Jones G, Parsons S, Limpens HJGA. Bat echolocation research: tools, techniques and analysis. Austin, Texas: Bat Conservation International; 2004.

[18] Kalcounis-Rueppell MC, Metheny JD, Vonhof MJ. Production of ultrasonic vocalizations by *Peromyscus* mice in the wild. Front Zool. 2006;3: 3 doi: 10.1186/1742-9994-3-3 16507093 10.1186/1742-9994-3-3 PMC1524959

[19] Simon M, Nuuttila H, Reyes-Zamudio MM, Ugarte F, Verfub U, Evans PGH. Passive acoustic monitoring of bottlenose dolphin and harbour porpoise, in Cardigan Bay, Wales, with implications for habitat use and partitioning. J Mar Biol Assoc U.K. 2010;90: 1539–1545. doi: 10.1017/S0025315409991226

[20] Heinicke S, Kalan AK, Wagner OJJ, Mundry R, Lukashevich H, Kühl HS. Assessing the performance of a semi-automated acoustic monitoring system for primates. Methods Ecol Evol. 2015;6: 753–763. doi: 10.1111/2041-210X.12384

[21] Zsebők S, Czabán D, Farkas J, Siemers BM, von Merten S. Acoustic species identification of shrews: Twittering calls for monitoring. Ecol Inform. 2015;27: 1–10. doi: 10.1016/j.ecoinf.2015.02.002

[22] Marcoux M, Ferguson SH, Roy N, Bedard JM, Simard Y. Seasonal marine mammal occurrence detected from passive acoustic monitoring in Scott Inlet, Nunavut, Canada. Polar Biol. 2017;40: 1127–1138. doi: 10.1007/s00300-016-2040-9

[23] Walters CL, Freeman R, Collen A, Dietz C, Brock Fenton M, Jones G, et al A continental-scale tool for acoustic identification of European bats. J Appl Ecol. 2012;49: 1064–1074. doi: 10.1111/j.1365-2664.2012.02182.x

[24] Zamora-Gutierrez V, Lopez-Gonzalez C, MacSwiney Gonzalez MC, Fenton B, Jones G, Kalko EKV, et al Acoustic identification of Mexican bats based on taxonomic and ecological constraints on call design. Methods Ecol Evol. 2016;7: 1082–1091. doi: 10.1111/2041-210X.12556

[25] Aubin T, Mathevon N, Staszewski V, Boulinier T. Acoustic communication in the Kittiwake *Rissa tridactyla*: potential cues for sexual and individual signatures in long calls. Polar Biol. 2007;30: 1027–1033. doi: 10.1007/s00300-007-0262-6

[26] Puechmaille SJ, Borissov IM, Zsebok S, Allegrini B, Hizem M, Kuenzel S, et al Female mate choice can drive the evolution of high frequency echolocation in bats: A case study with *Rhinolophus mehelyi*. PLOS ONE. 2014;9: e103452 doi: 10.1371/journal.pone.0103452 25075972 10.1371/journal.pone.0103452 PMC4116191

[27] Schuchmann M, Puechmaille S, Martin Siemers B. Horseshoe bats recognise the sex of conspecifics from their echolocation calls. Acta Chiropt. 2012;14: 161–166. doi: 10.3161/150811012X654376

[28] Jones G, Gordon T, Nightindale J. Sex and age differences in the echolocation calls of the lesser horseshoe bat, *Rhinolophus hipposideros*. Mammalia. 1992;56: 189–193.

[29] Peake TM, McGregor PK, Smith KW, Tyler G, Gilbert G, Green RE. Individuality in Corncrake *Crex crex* vocalizations. Ibis. 1998;140: 120–127. doi: 10.1111/j.1474-919X.1998.tb04548.x

[30] Fischer J, Kitchen DM, Seyfarth RM, Cheney DL. Baboon loud calls advertise male quality: acoustic features and their relation to rank, age, and exhaustion. Behav Ecol Sociobiol. 2004;56: 140–148. doi: 10.1007/s00265-003-0739-4

[31] Reby D, McComb K. Anatomical constraints generate honesty: acoustic cues to age and weight in the roars of red deer stags. Anim Behav. 2003;65: 519–530. doi: 10.1006/anbe.2003.2078

[32] Charlton BD, Zhihe Z, Snyder RJ. The information content of giant panda, *Ailuropoda melanoleuca*, bleats: acoustic cues to sex, age and size. Anim Behav. 2009;78: 893–898. doi: 10.1016/j.anbehav.2009.06.029

[33] Maluleke T, Jacobs DS, Winker H. Environmental correlates of geographic divergence in a phenotypic trait: A case study using bat echolocation. Ecol Evol. 2017;7: 7347–7361. doi: 10.1002/ece3.3251 28944021 10.1002/ece3.3251 PMC5606872

[34] Laiolo P, Rolando A, Delestrade A, de Sanctis A. Geographical variation in the calls of the choughs. The Condor. 2001;103: 287–297. doi: 10.1650/0010-5422(2001)103[0287:GVITCO]2.0.CO;2

[35] Puechmaille SJ, Gouilh MA, Piyapan P, Yokubol M, Mie KM, Bates PJ, et al The evolution of sensory divergence in the context of limited gene flow in the bumblebee bat. Nat. Commun. 2011;2: 573 doi: 10.1038/ncomms1582 22146392 10.1038/ncomms1582 PMC3247819

[36] Volodin IA, Volodina EV, Klenova AV, Matrosova VA. Gender identification using acoustic analysis in birds without external sexual dimorphism. Avian Res. 2015;6: 20.

[37] Francis CM, Habersetzer J. Interspecific and intraspecific variation in echolocation call frequency and morphology of horseshoe bats, *Rhinolophus* and *Hipposideros* In: Kunz TH, Racey PA, editors. Bat Biology and Conservation. Washington: Smithsonian Institution Press; 1998 pp. 169–179.

[38] Issel W. Ökologische Untersuchungen an der Kleinen Hufeisennase (*Rhinolophus hipposideros* Bechstein) im mittleren Rheinland und unterem Altmühltal. Zool Jb Sys. 1950;79: 71–86.

[39] Gaisler J. The ecology of lesser horseshoe bat (*Rhinolophus hipposideros* Bechstein, 1800) in Czechoslovakia, part I. Vest Cls Spol Zool. 1963;27: 211–233.

[40] Bontadina F, Schofield H, Naef-Daenzer B. Radio-tracking reveals that lesser horseshoe bats (*Rhinolophus hipposideros*) forage in woodland. J Zool. 2002;258: 281–290.

[41] Whitby MD, Carter TC, Britzke ER, Bergeson SM. Evaluation of mobile acoustic techniques for bat population monitoring. Acta Chiropt. 2014;16: 223–230. doi: 10.3161/150811014X683417

[42] Jones G, Siemers BM. The communicative potential of bat echolocation pulses. J Comp Physiol A. 2011;197: 447–457. doi: 10.1007/s00359-010-0565-x 20686895 10.1007/s00359-010-0565-x

[43] Gillam E, Fenton MB. Roles of acoustic social communication in the lives of bats In: Fenton MB, Grinnell AD, Popper AN, Fay RR, editors. Bat bioacoustics. New York, NY: Springer New York; 2016 pp. 117–139.

[44] Scrucca L, Fop M, Murphy TB, Raftery AE. mclust 5: clustering, classification and density estimation using Gaussian finite mixture models. The R Journal. 2016 8/1: 205–233. PMC5096736 27818791

[45] Kruschke JK. Doing Bayesian Data Analysis A Tutorial with R, JAGS, and Stan. 2nd ed Academic Press/Elsevier; 2015.

[46] Barataud M. Ecologie acoustique des chiroptères d’Europe Identification des espèces, étude de leurs habitats et comportements de chasse. Mèze & Paris: Coédition Biotope; 2012.

[47] Dool SE, Puechmaille SJ, Kelleher C, McAney K, Teeling EC. The effects of human-mediated habitat fragmentation on a sedentary woodland-associated species (*Rhinolophus hipposideros*) at its range margin. Acta Chiropt. 2016;18: 377–393. doi: 10.3161/15081109ACC2016.18.2.006

[48] Wimmer, B, Kugelschafter, K. Akustische Erfassung von Fledermäusen in unterirdischen Quartieren [Internet]. 2017. http://www.fledermausrufe.de/Ortungs_Sozialrufe_unterirdischeQuartiere.pdf

[49] Lenormand M, Jabot F, Deffuant G. Adaptive approximate Bayesian computation for complex models. Comput Stat. 2013;28: 2777–2796. doi: 10.1007/s00180-013-0428-3

[50] Jabot F, Faure T, Dumoulin N, Albert C. EasyABC: Efficient Approximate Bayesian Computation sampling schemes [Internet]. 2015. https://CRAN.R-project.org/package=EasyABC

[51] R Development Core Team. R: a language and environment for statistical computing [Internet]. Vienna (Austria): R Foundation for Statistical Computing; 2017 http://cran.r-project.org

[52] Meredith M, Kruschke J. HDInterval: Highest (Posterior) Density Intervals. R package version 0.1.3. https://CRAN.R-project.org/package=HDInterval

[53] Andrews MM, Hodnett AM, Andrews PT. Social activity of Lesser horseshoe bats (*Rhinolophus hipposideros*) at nursery roosts and a hibernaculum in North Wales, U.K. Acta Chiropt. 2017;19: 161–174. doi: 10.3161/15081109ACC2017.19.1.013

[54] Puechmaille SJ, Petit EJ. Empirical evaluation of non-invasive capture—mark—recapture estimation of population size based on a single sampling session. J Appl Ecol. 2007;44: 843–852. doi: 10.1111/j.1365-2664.2007.01321.x

[55] Puechmaille SJ, Mathy G, Petit EJ. Good DNA from bat droppings. Acta Chiropt. 2007;9: 269–276. doi: 10.3161/1733-5329(2007)9[269:GDFBD]2.0.CO;2

[56] Covarrubias-Pazaran G, Diaz-Garcia L, Schlautman B, Salazar W, Zalapa J. Fragman: an R package for fragment analysis. BMC Genet. 2016;17: 1–8. 27098093 10.1186/s12863-016-0365-6 PMC4839125

[57] Jiang T, Wu H, Feng J. Patterns and causes of geographic variation in bat echolocation pulses. Integr Zool. 2015;10: 241–256. doi: 10.1111/1749-4877.12129 25664901 10.1111/1749-4877.12129

[58] López-Baucells A, Torrent L, Rocha R, Pavan AC, Bobrowiec PED, Meyer CFJ. Geographical variation in the high-duty cycle echolocation of the cryptic common mustached bat *Pteronotus cf*. *rubiginosus* (Mormoopidae). Bioacoustics. 2017; doi: 10.1080/09524622.2017.1357145

[59] Leonard ML, Horn AG. Crowing in relation to status in roosters. Anim Behav. 1995;49: 1283–1290. doi: 10.1006/anbe.1995.0160

[60] Colleye O, Frederich B, Vandewalle P, Casadevall M, Parmentier E. Agonistic sounds in the skunk clownfish *Amphiprion akallopisos*: size-related variation in acoustic features. J Fish Biol. 2009;75: 908–916. doi: 10.1111/j.1095-8649.2009.02316.x 20738587 10.1111/j.1095-8649.2009.02316.x

[61] Jones G. Acoustic signals and speciation: the roles of natural and sexual selection in the evolution of cryptic species. Advances in the Study of Behavior. 1997;26: 317–354.

[62] Kingston T, Lara MC, Jones G, Akbar Z, Kunz TH, Schneider CJ. Acoustic divergence in two cryptic *Hipposideros* species: a role for social selection? Proc Biol Sci. 2001;268: 1381 doi: 10.1098/rspb.2001.1630 11429138 10.1098/rspb.2001.1630 PMC1088752

[63] Bickford D, Lohman DJ, Sodhi NS, Ng PKL, Meier R, Winker K, et al Cryptic species as a window on diversity and conservation. Trends Ecol Evol. 2007;22: 148–155. doi: 10.1016/j.tree.2006.11.004 17129636 10.1016/j.tree.2006.11.004

[64] Angulo A, Reichle S. Acoustic signals, species diagnosis, and species concepts: the case of a new cryptic species of *Leptodactylus* (Amphibia, Anura, Leptodactylidae) from the Chapare region, Bolivia. Biol J Linn Soc. 2008;152: 59–77. doi: 10.1111/j.1096-3642.2007.00338.x

[65] Hughes AC, Satasook C, Bates PJJ, Soisook P, Sritongchuay T, Jones G, et al Echolocation call analysis and presence-only modelling as conservation monitoring tools for Rhinolophoid bats in Thailand. Acta Chiropt. 2010;12: 311–327. doi: 10.3161/150811010X537891

[66] Henry CS, Brooks SJ, Johnson JB, Mochizuki A, Duelli P. A new cryptic species of the *Chrysoperla carnea* group (Neuroptera: Chrysopidae) from western Asia: parallel speciation without ecological adaptation. Syst Entomol. 2014;39: 380–393. doi: 10.1111/syen.12061

[67] Ancillotto L, Mori E, Sozio G, Solano E, Bertolino S, Russo D. A novel approach to field identification of cryptic *Apodemus* wood mice: calls differ more than morphology. Mam Rev. 2017;47: 6–10. doi: 10.1111/mam.12076

[68] Safi K, König B, Kerth G. Sex differences in population genetics, home range size and habitat use of the parti-colored bat (*Vespertilio murinus*, Linnaeus 1758) in Switzerland and their consequences for conservation. Biol Conserv. 2007;137: 28–36. doi: 10.1016/j.biocon.2007.01.011

[69] Griffiths ET, Keen SC, Lanzone M, Farnsworth A. Can nocturnal flight calls of the migrating songbird, American redstart, encode sexual dimorphism and individual identity? PLOS ONE. 2016;11: e0156578 doi: 10.1371/journal.pone.0156578 27284697 10.1371/journal.pone.0156578 PMC4902225

[70] Azam C, Le Viol I, Julien J-F, Bas Y, Kerbiriou C. Disentangling the relative effect of light pollution, impervious surfaces and intensive agriculture on bat activity with a national-scale monitoring program. Landsc Ecol. 2016;31: 2471–2483. doi: 10.1007/s10980-016-0417-3

[71] Cvikel N, Levin E, Hurme E, Borissov I, Boonman A, Amichai E, et al On-board recordings reveal no jamming avoidance in wild bats. Proc R Soc B. 2015;282: 20142274 doi: 10.1098/rspb.2014.2274 25429017 10.1098/rspb.2014.2274 PMC4262180

[72] Götze S, Koblitz JC, Denzinger A, Schnitzler H-U. No evidence for spectral jamming avoidance in echolocation behavior of foraging pipistrelle bats. Sci Rep. 2016;6: 30978 doi: 10.1038/srep30978 27502900 10.1038/srep30978 PMC4977515

