# Supplementary figures and images for: Using Approximate Bayesian Computation to infer sex ratios from acoustic data
Source: PLoS One. 2018 Jun 21;13(6):e0199428. doi: 10.1371/journal.pone.0199428 (PMC6013104; doi:10.1371/journal.pone.0199428)

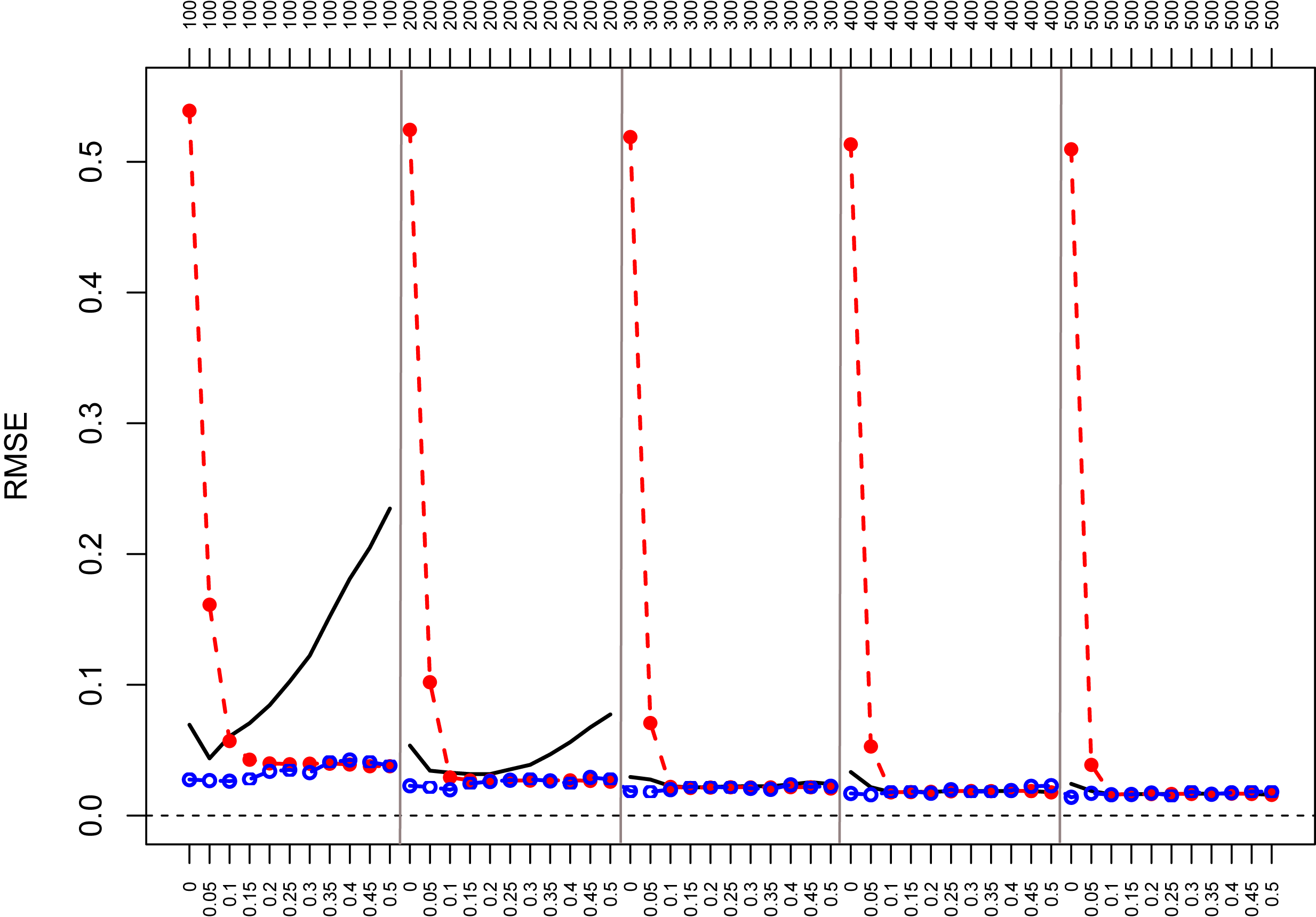

Supplement: S1 Fig — Black line: RMSE with the number of groups determined by the model. Red dotted line: RMSE with the number of groups set to two by the user. Sample size is from n = 100 to 500 (in steps of 100), as indicated above the graph. Blue circles: RMSE obtained with the ABC approach. (TIF) [file pone.0199428.s001.tif]

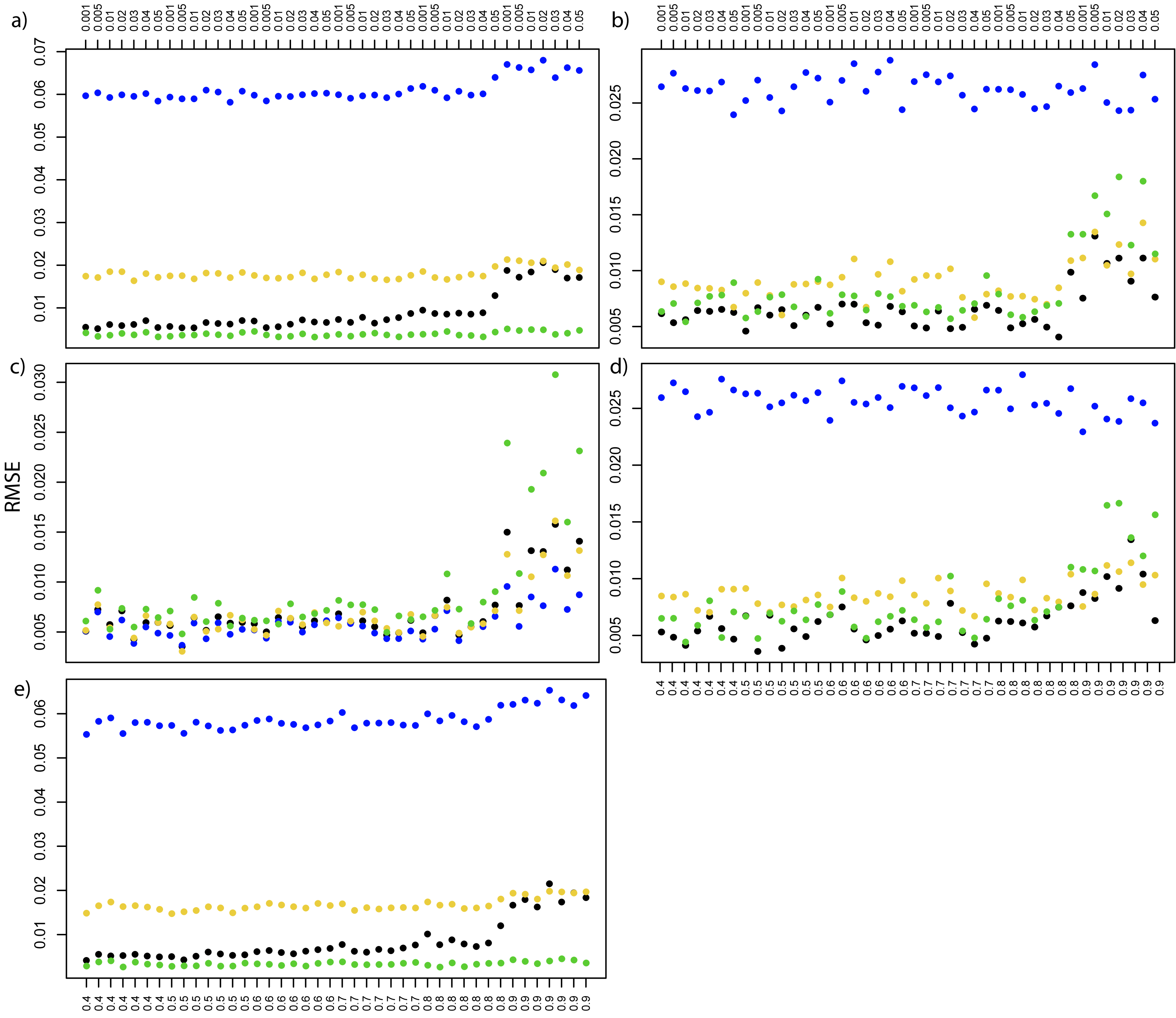

Supplement: S2 Fig — RMSEs of estimates obtained with a sample size of 5000 calls for different parameter combinations of the ABC algorithm and different sex ratio; a) POM = 0, b) POM = 0.25, c) POM = 0.5, d) POM = 0.75 and e) POM = 1. The values of p_acc_min and alpha used are presented above and below the graphs respectively. Consistently low root mean square error (RMSE) was obtained for nb_simul = 1000, alpha = 0.4, p_acc_min = 0.01. Black: ABC, blue: 95% exclusion method, yellow: 99% exclusion method, green: 99.9% exclusion method. (TIF) [file pone.0199428.s002.tif]
