# Supplementary material for: Using Approximate Bayesian Computation to infer sex ratios from acoustic data
Source: PLoS One. 2018 Jun 21;13(6):e0199428. doi: 10.1371/journal.pone.0199428 (PMC6013104; doi:10.1371/journal.pone.0199428)
Supplement: S1 Table — (PDF) [file pone.0199428.s003.pdf]

**Table S1. Sampling period and location (rounded to two decimals) of the four *Rhip* colonies from which the proportion of males was estimated.**

| | Sampling period | | Coordinates | |
|---|---|---|---|---|
| ID | Genetic | Acoustic | X | Y |
| Thu22 | 19.06.-30.06.15 | 20.05.-19.06.15 | 50.95 | 11.55 |
| Thu26 | 19.06.-01.07.15 | 19.05.-19.06.15 | 50.91 | 11.54 |
| Thu47 | 31.05.-16.06.16 | 17.06.-10.08.16 | 50.89 | 11.02 |
| Thu35 | 17.06.-08.07.16 | 17.06.-04.07.16 | 50.82 | 10.33 |
