# Supplementary material for: Using Approximate Bayesian Computation to infer sex ratios from acoustic data
Source: PLoS One. 2018 Jun 21;13(6):e0199428. doi: 10.1371/journal.pone.0199428 (PMC6013104; doi:10.1371/journal.pone.0199428)
Supplement: S3 File — (PDF) [file pone.0199428.s007.pdf]

S3 File: Testing the performance of the mclust package for estimating the proportion of males

The contributed R package mclust (version 5.3) was used to perform model-based clustering of simulated datasets (mimicking echolocation calls as detailed in the 'Simulated data set' section of the Material and Methods). Simulated datasets included different numbers of calls, from 100 to 500 in steps of 100. The proportion of males in the simulated datasets varied between 0 and 0.5 in steps of 0.05. A total of 10,000 simulated datasets was tested for each combination of the number of calls and proportion of males (55 possible combinations). We used the function 'Mclust' of the package for parameter estimation via the EM algorithm for normal (univariate) mixture models with equal variance. Variance was set as equal as it was indeed equal in the simulated datasets but also to allow direct comparison with the results from the ABC approach which also assumed equal variance (see section 'Inferring sex ratios with ABC' in the main text). The number of mixture components (clusters) was either *1)* determined based on optimality according to Bayesian Information Criterion (BIC) or *2)* manually forced to two components (G=2). Both options were investigated to compare their performance. Performance was estimated using the root mean-squared error (RMSE, detailed in the 'Methods performance' section of the main text).
