# Supplementary material for: Using Approximate Bayesian Computation to infer sex ratios from acoustic data
Source: PLoS One. 2018 Jun 21;13(6):e0199428. doi: 10.1371/journal.pone.0199428 (PMC6013104; doi:10.1371/journal.pone.0199428)
Supplement: S4 File — (PDF) [file pone.0199428.s008.pdf]

S4 File: What are recordings in our study?

Our Anabat SD2 bat detector recorded a continuous stream of data that was broken up into files for analysis. To do so, we used the program CFCread (Titley) with the three parameters as recommended in the documentation provided with the software (documentation 'CFCread File Saving Limitations.docx'). These are: Smooth (default to 50), MaxTBC (default to 5), Min Line Length (default to 5). In this situation, a pulse is recognised as a potential bat call if it meets certain criteria as detailed in the documentation:

1) The dots within the call must be smoothly connected, which means that each dot must be close to the average of the dots either side of it. How close is decided by the Smooth parameter which was set by default to 50.

2) A sequence of dots which are sufficiently smoothly connected will be considered a bat call if it has a length greater than the Min Line Length parameter. This parameter specifies the number of dots which must be smoothly connected.

A file will only be saved if at least two such calls are detected within the time set by MaxTBC (in seconds). If a file is to be saved, the file will always start from the beginning of the first pulse detected. When it ends depends on the following criteria:

1) If the buffer is filled, the file will be saved immediately. The buffer can hold up to 16384 data points, so this criteria is not commonly met. But noisy files, or files containing many bats could reach this limit. If files contain lots of data and are shorter than 15 seconds, it is likely to be for this reason. Files which have met this criterion will always be large - usually at least 16K.

2) If there has been no data for MaxTBC seconds, then the file will be saved at this point. This criterion can result in files of short duration, potentially much shorter than 15 seconds.

3) If the time since the start of the first potential bat call is greater than 15 seconds, the file will be saved at that point. This ensures a file can never be longer than 15 seconds.
