# Supplementary material for: Using Approximate Bayesian Computation to infer sex ratios from acoustic data
Source: PLoS One. 2018 Jun 21;13(6):e0199428. doi: 10.1371/journal.pone.0199428 (PMC6013104; doi:10.1371/journal.pone.0199428)
Supplement: S6 File — (PDF) [file pone.0199428.s010.pdf]

S6 File: Modified protocol for extraction of DNA from bat faeces with the Macherey Nagel NucleoSpin® Plant II extraction kit

Preparation: As detailed in the manual provided by the manufacturer (http://www.mn-net.com/Portals/8/attachments/Redakteure_Bio/Protocols/Genomic%20DNA/UM_gDNAPlant_NS8PlantII.pdf).

Homogenization: Place faecal pellets into the tube strips, one pellet per tube. Add two 2 mm diameter glass beads and homogenize in a homogenizer (here, Qiagen Tissue Lyser II) by shaking for two times 20 seconds at 20 Hz. Turn the plates in between homogenization steps. Centrifuge the powdered samples at 6000 g for 5 minutes.

Lysis: Add 425 µl of lysis buffer PL2 to each sample. Shake by hand for 30 seconds. Centrifuge for 30 seconds at 750 g. Incubate the rack of tube strips with the samples on a thermoshaker at 60°C for 6 hours (medium shaking speed). Add 105 µl of lysis buffer PL3 to each sample. Shake by hand for 30 seconds. Remove the bottom of the tube strip racks and incubate on ice for 15 minutes. Centrifuge at 6000 g for 20 minutes.

Binding: Pre-dispense 450 µl of buffer PC into each well of the MN-square-well-block. Add 340 µl of cleared lysate supernatant. Mix three times by pipetting up and down. Transfer the mix onto the binding plate stacked on a MN-square-well-block. Seal binding plate with provided gas-permeable foil. Centrifuge at 6000 g for 5 minutes.

Washing: Add 400 µl PW1 (prepared as indicated in manual), seal with gas permeable foil and centrifuge at 6000 g for 2 minutes. Stack binding plate onto new MN-square-well-block. Add 700 µl PW2, seal with gas permeable foil and centrifuge at 6000 g for 2 minutes. Stack binding plate onto new MN-square-well-block. Wash again with 700 µl PW2.

Elution: Stack the binding plate on the storage plate (here, 96 well Abgene Storage Plates, Thermo Fischer). Dispense directly onto the membrane 75µl PE pre-heated to 70°C. Incubate at 70°C for 2 minutes. Centrifuge at 6000 g for 2 minutes. Repeat the elution step with another 75 µl of elution buffer, without the incubation step. Seal and freeze the plate at -20°C for storage.
